# Preoperative ultrasound combined with routine blood tests in predicting the malignant risk of pancreatic cystic neoplasms

**DOI:** 10.20892/j.issn.2095-3941.2022.0258

**Published:** 2022-11-01

**Authors:** Xiuchao Wang, Junjin Wang, Xi Wei, Lihui Zhao, Bo Ni, Zekun Li, Chuntao Gao, Song Gao, Tiansuo Zhao, Jian Wang, Weidong Ma, Xiao Hu, Jihui Hao

**Affiliations:** 1 Department of Pancreatic Cancer, Tianjin Medical University Cancer Institute & Hospital, National Clinical Research Center for Cancer, Key Laboratory of Cancer Prevention and Therapy, Tianjin, Tianjin’s Clinical Research Center for Cancer, Tianjin 300060, China; 2 Department of Diagnostic and Therapeutic Ultrasonography, Tianjin Medical University Cancer Institute & Hospital, National Clinical Research Center for Cancer, Key Laboratory of Cancer Prevention and Therapy, Tianjin, Tianjin’s Clinical Research Center for Cancer, Tianjin 300060, China; 3 Department of Hepatobiliary and Pancreatic Surgery, The Affiliated Hospital of Qingdao University, Qingdao 266000, China

**Keywords:** Pancreatic cystic neoplasms, malignancy prediction, nomogram, ultrasound, blood routine

## Abstract

**Objective:**

Accurate preoperative identification of benign or malignant pancreatic cystic neoplasms (PCN) may help clinicians make better intervention choices and will be essential for individualized treatment.

**Methods:**

Preoperative ultrasound and laboratory examination findings, and demographic characteristics were collected from patients. Multiple logistic regression was used to identify independent risk factors associated with malignant PCN, which were then included in the nomogram and validated with an external cohort. The Net Reclassification Index (NRI) and Integrated Discrimination Improvement (IDI) were calculated to evaluate the improvement in the predictive power of the new model with respect to that of a combined imaging and tumor marker prediction model.

**Results:**

Malignant PCN were found in 83 (40.7%) and 33 (38.7%) of the model and validation cohorts, respectively. Multivariate analysis identified age, tumor location, imaging of tumor boundary, blood type, mean hemoglobin concentration, neutrophil-to-lymphocyte ratio, carbohydrate antigen 19-9, and carcinoembryonic antigen as independent risk factors for malignant PCN. The calibration curve indicated that the predictions based on the nomogram were in excellent agreement with the actual observations. A nomogram score cutoff of 192.5 classified patients as having low *vs.* high risk of malignant PCN. The model achieved good C-statistics of 0.929 (95% CI 0.890–0.968, *P* < 0.05) and 0.951 (95% CI 0.903–0.998, *P* < 0.05) in predicting malignancy in the development and validation cohorts, respectively. NRI = 0.268; IDI = 0.271 (*P* < 0.001 for improvement). The DCA curve indicated that our model yielded greater clinical benefits than the comparator model.

**Conclusions:**

The nomogram showed excellent performance in predicting malignant PCN and may help surgeons select patients for detailed examination and surgery. The nomogram is freely available at https://wangjunjinnomogram.shinyapps.io/DynNomapp/.

## Introduction

In recent years, with increasing development and application of imaging technology, and greater public health awareness, the detection rate of pancreatic cystic neoplasms (PCN) has been increasing annually^1^. The most common types of PCN are serous cystic neoplasms (SCN), mucinous cystic neoplasms (MCN), and intraductal papillary mucinous neoplasms (IPMN). These PCN are considered to develop from low-grade dysplasia to high-grade dysplasia, and then to invasive cancer^2,3^. After aggressive disease develops, the survival outcomes are similar to those of traditional pancreatic cancer. As the detection rate and the potential risk of malignant transformation increase each year, the diagnosis and treatment of PCN are receiving increasing attention from clinicians. Because surgery itself increases patients’ mortality and morbidity, optimal surveillance for patients with PCN is becoming an increasingly common clinical problem.

The primary goal of preoperative PCN diagnosis is to prevent malignancies and avoid unnecessary surgery. Clinicians are frequently challenged by differential diagnosis and subsequent management of the various types of lesions in the pancreas, thus potentially leading to overtreatment or delayed treatment. Therefore, careful diagnosis is required to determine whether a lesion is benign or malignant, or has malignant potential, and clinicians must assess whether patients are at high risk or low risk before surgery. At present, most physicians believe that resection of high-grade intraepithelial neoplastic tumors has important therapeutic value. They can choose minimally invasive and functional surgery, and avoid late chemotherapy and radiotherapy, thus greatly improving patient prognosis and quality of life. However, on the basis of analysis of postoperative pathological results, less than half of all patients who undergo pancreatectomy have high-risk pathology, thus suggesting that current methods for resection and evaluation of pancreatic cystic tumors are imperfect. Excessive surgical treatment may result, thus harming patients and consuming many medical resources.

Currently, the early detection of PCN is mainly incidental through ultrasound during physical examinations. Most patients undergo further investigation such as enhanced computed tomography (CT) and/or enhanced magnetic resonance imaging (MRI), endoscopic ultrasound-guided fine-needle aspiration biopsy, and surgical resection after a lesion is detected^4^. Imaging examinations can determine the location, shape, size, and number of tumors, as well as the presence of solid components, thus serving as an important tool to determine PCN benignity or malignancy preoperatively. However, most studies have shown that no single imaging test is sufficiently accurate to distinguish benign from malignant PCN. A systematic review has shown that the accuracy of CT and MRI in distinguishing benign from malignant PCN is 71%–80% and 55%–76%, respectively^5^. Although cystic fluid analysis for the diagnosis of MCN and IPMN provides high accuracy^6^, no guidelines recommend the use of these methods, and fine-needle aspiration of cystic fluid is theoretically associated with the risk of tumor implantation and relatively high financial pressure on patients; therefore, this method is controversial and is performed in only several centers.

Blood is rich in tumor-associated biomarkers, and each molecule in the blood has its own potential diagnostic, prognostic, and surveillance value for cancer. Some blood inflammatory indicators such as the neutrophil-to-lymphocyte ratio (NLR), platelet-to-lymphocyte ratio (PLR), and lymphocyte-to-monocyte ratio^7–11^ can be used as markers of the systemic inflammatory response and as independent predictors of prognosis in various malignancies including pancreatic cancer. Platelets can provide relevant information regarding cancer^12^. The non-O blood type is also considered a risk factor for malignant tumors^13,14^.

Abdominal ultrasound is simple and inexpensive, can detect cystic lesions of the pancreas and differentiate them from solid lesions, and is currently used as a primary screening tool for PCN in clinical practice. In this study, we sought to combine ultrasound imaging, laboratory examination, and patient demographic characteristics to develop and externally validate a new model. This model can accurately predict the risk of malignancy in PCN in an easy, safe, and economical manner, and may help clinicians select appropriate interventions at the time of the initial patient diagnosis.

## Materials and methods

The study was approved by the Ethics Committee of the Tianjin Medical University Cancer Institute & Hospital (Approval No. bc2019063). All patients provided written informed consent approving the use of their data for research purposes.

### Indications for surgery

Surgical resection was performed for patients who met the following indications. First, pathological biopsy suggesting cellular carcinoma, with invasive carcinoma or a clinically suspected malignant cystic tumor, was an absolute indication for surgical intervention. Second, patients with tumor-associated obstructive jaundice or recurrent episodes of acute pancreatitis as well as abdominal pain, abdominal distension, nausea, vomiting, or diarrhea were recommended to undergo surgical resection. Third, surgical resection was recommended for patients with excessive tumor size (> 3 cm), a rapid tumor growth rate (> 5 mm/year), imaging showing cysts communicating with the main pancreatic duct, main pancreatic duct dilatation ≥ 1 cm, and abnormally elevated tumor markers.

### Patient selection

The data for patients admitted to Tianjin Medical University Cancer Institute & Hospital between June 2018 and June 2021 with pathology confirmed as PCN after surgery were retrospectively analyzed to form a development cohort. The main patient inclusion criteria were as follows: (1) primary patients with pathologically confirmed IPMN, MCN, or SCN after surgical treatment and (2) patients with complete clinical information, including past medical history, laboratory tests, imaging tests, and postoperative pathological examinations.

The exclusion criteria included (1) patients with malignant tumors other than pancreatic cancer, (2) patients who had undergone chemotherapy, radiotherapy, or other tumor-related treatments, (3) patients with other pancreatic diseases or who had undergone pancreatic-related surgeries, and (4) patients with incomplete clinical information.

The 86 patients with PCN diagnosed at the Affiliated Hospital of Qingdao University from 2018 to 2020 were included in the validation cohort.

SPN were not included in this study, because of the clear difference in imaging features and demographic characteristics with respect to those of the other 3 types, and the clear indication for surgery. MCN and IPMN are considered to form malignant cystic tumors more readily, whereas SCN are mostly benign tumors. However, SCN were included in our study because they have diverse imaging features and are prone to misdiagnosis as malignant tumors. According to the classification proposed by Kimura et al.^15^, SCN can be classified into 4 types: microcystic, macrocystic, mixed, and solid. Macrocystic SCN can sometimes appear unicystic without separation or oligocystic with little separation, which is indistinguishable from mucinous cystadenoma. Mixed type SCN usually show a small cyst in the middle and a large cyst at the periphery. The cysts of solid SCN are small and often appear solid on imaging, the small cystic structures inside are difficult to detect. More than 70% of SCN are microcystic in nature, and their typical imaging features include stellate central scarring and spoke-like microsegments with lobulated periphery, thin walls, numerous microcysts forming a honeycomb section inside, and no communication with the pancreatic duct. However, several microcystic SCN have atypical clinical and imaging manifestations, which may be accompanied by pancreatic duct and/or bile duct dilatation, pancreatic parenchymal atrophy, suspicion of vascular invasion, invasion of adjacent organs, communication with the pancreatic duct, and other atypical imaging features^16,17^.

### Data collection

The clinical data collected mainly included baseline characteristics of patients, clinical manifestations, laboratory examinations, ultrasound imaging, and pathological diagnosis. Jaundice was defined by total serum bilirubin > 34.2 μmol/L, and a solid component was defined as a cystic area suggestive of a real component on preoperative ultrasound. Benign PCN were defined as cystic adenoma or low-/moderate-grade dysplasia (CIN I/II) confirmed by a pathological report in the postoperative histological section. Malignant PCN were defined as high-grade dysplasia (CIN III)/associated invasive carcinoma with PCN.

### Statistical analysis

Tumor benignity and malignancy were used as dependent variables, and the cutoff values for continuous variables were determined with the pROC package. The clinical data for patients in the development cohort were analyzed with Best Subsets Regression (BSR), Lasso regression, and univariate logistic regression. The variables screened by Lasso regression and those with *P* < 0.15 in the univariate analysis were subjected to backward stepwise regressions. The variables screened by the 3 approaches were included in the multi-factor regression model based on the Akaike information criterion to select the combination of variables with the smallest AIC. The variables with *P* < 0.05 in multiple logistic regression were used to develop the nomogram.

The discrimination ability of the nomogram was identified by application of the concordance index (C-index). The calibration curve was plotted to reflect the consistency between the observed results and the predicted probabilities. The score of each patient in the nomogram was calculated. A receiver operating characteristic (ROC) curve was plotted, and the area under the curve was calculated to assess the predictive efficacy of the model, and the optimal cutoff value was determined on the basis of the Youden index. To assess the utility of the model, we compared the nomogram with the common clinical imaging features assessed in combination with tumor markers; NRI and IDI were calculated; and decision curve analysis (DCA) was used to determine the clinical utility of the nomogram by quantifying the net benefit at different threshold probabilities. Finally, clinical data from the validation cohort were used to assess the accuracy, sensitivity, and specificity of the predictive model. All statistical analyses were performed in SPSS version 26.0 (IBM) and R version 4.1.0 (http://www.r-project.org/).

## Results

### Demographics

A total of 204 patients with PCN were included in the development cohort, including 88 cases of IPMN (43.1%), 30 cases of MCN (14.7%), and 86 cases of SCN (42.2%). The average age of the included patients was 56.5 years, ranging from 20 to 76 years of age. Among them, 122 cases were in women, and 82 cases were in men. The pathological types included 121 cystadenomas/CIN I/II and 83 cases of CIN III/invasive carcinoma associated with PCN. The validation cohort consisted of 86 patients diagnosed with PCN at the Affiliated Hospital of Qingdao University from 2018 to 2020, including 27 cases of SCN (31.4%), 28 cases of MCN (32.6%), and 31 cases of IPMN (36.0%). The mean age of the validation cohort was 60 years. The pathological types included 53 cystadenomas/CIN I/II and 33 cases of CIN III/invasive carcinoma associated with PCN.

### Univariate regression analysis

**Figure 1:**
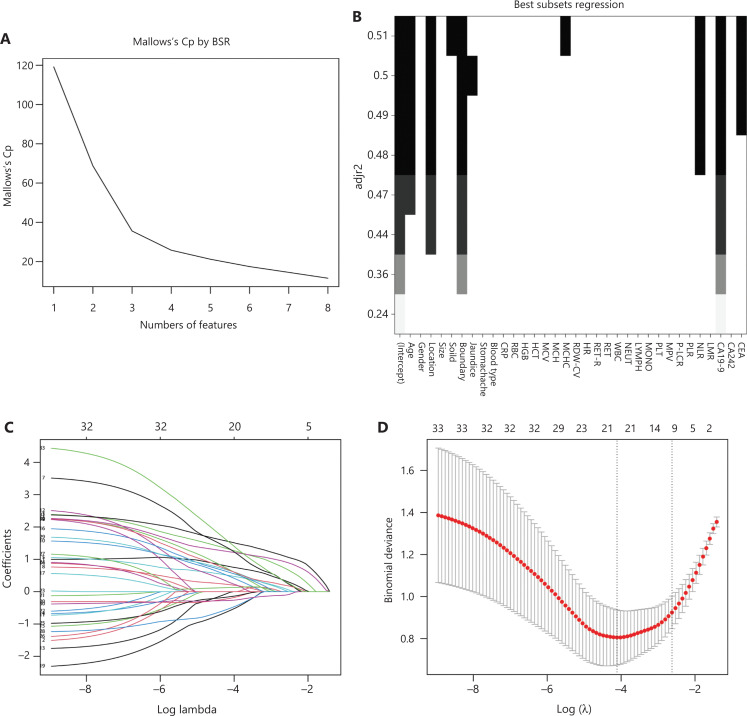
Screening of model variables. Variables were screened with Best Subsets Regression (BSR) (A, B; AIC = 151.3), Lasso regression (C, D; AIC = 152.27), and univariate regression and multiple logistic regression (AIC = 144.02).

**Figure 2:**
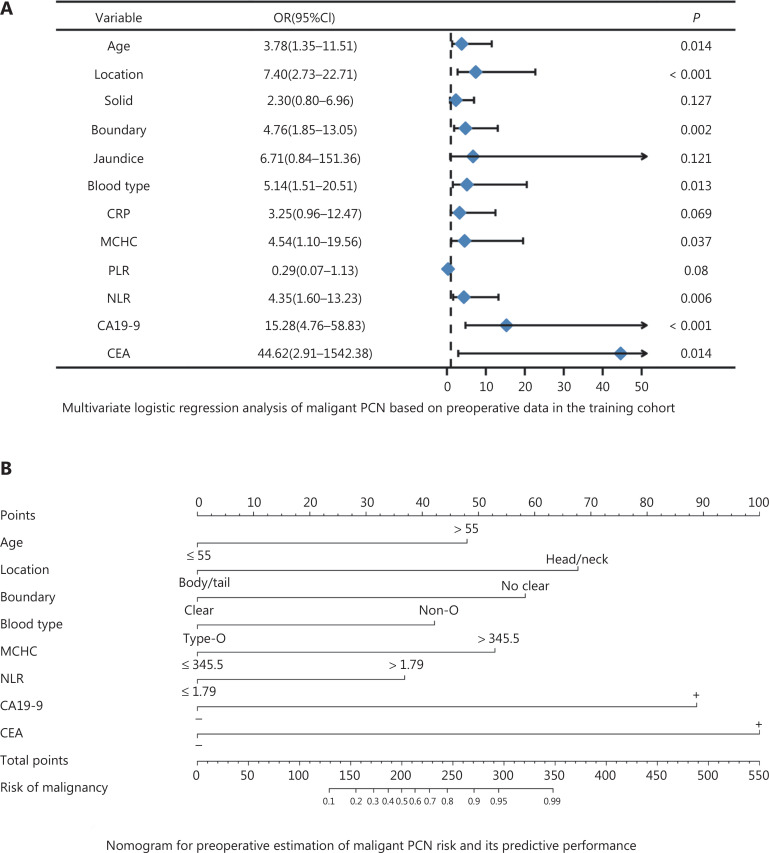
(A) Multivariate analysis revealing that increased CA19-9, increased CEA, increased NLR, increased MCHC, non-O blood group, age > 55 years, cysts located in the head or neck of the pancreas, and an unclear cyst imaging border were independent predictors of PCN malignancy. (B) Nomogram for estimating the risk of malignancy in PCN. The nomogram is used to find the position of each variable on the corresponding axis, a vertical line is drawn to the points axis to obtain the value of the corresponding variable, the points are added for all variables, and then a line is drawn from the total points axis to determine the probability of a malignant tumor at the lower part of the nomogram.

**Table 1** presents the results of univariate regression of variables in the model cohort. BSR, Lasso regression, and multiple-factor backward stepwise regression were performed to screen variables in the model cohort, and the results are displayed in **Figure 1**. Multivariate analysis identified age, location, boundary, blood type, MCHC, NLR, CA19-9, and CEA as independent predictors of malignant PCN (**Figure 2A**).

**Table 1:** Univariate logistic regression analysis based on preoperative data in the model cohort

Variable (%)	Level	Overall (%)	Benign (%)	Malignant (%)	OR (95% CI)	*P*
*n*	–	204	121	83	–	–
Age (years)	≤ 55	87 (42.6)	69 (57.0)	18 (21.7)	1.42 (1.25–1.61)	< 0.001*
	> 55	117 (57.4)	52 (43.0)	65 (78.3)	–	–
Gender	Female	122 (59.8)	84 (69.4)	38 (45.8)	1.27 (1.11–1.45)	0.001*
	Male	82 (40.2)	37 (30.6)	45 (54.2)	–	–
Location	Body/tail	111 (54.4)	84 (69.4)	27 (32.5)	1.43 (1.26–1.63)	< 0.001*
	Head/neck	93 (45.6)	37 (30.6)	56 (67.5)	–	–
Size (cm)	≤ 2.75	158 (77.5)	97 (80.2)	61 (73.5)	1.1 (0.93–1.29)	0.265
	> 2.75	46 (22.5)	24 (19.8)	22 (26.5)	–	–
Solid	No	79 (38.7)	62 (51.2)	17 (20.5)	1.37 (1.2–1.56)	< 0.001*
	Yes	125 (61.3)	59 (48.8)	66 (79.5)	–	–
Boundary	Clear	100 (49.0)	83 (68.6)	17 (20.5)	1.59 (1.41–1.79)	< 0.001*
	Blurred	104 (51.0)	38 (31.4)	66 (79.5)	–	–
Jaundice	No	187 (91.7)	120 (99.2)	67 (80.7)	1.79 (1.42–2.26)	< 0.001*
	Yes	17 (8.3)	1 (0.8)	16 (19.3)	–	–
Stomachache	No	109 (53.4)	75 (62.0)	34 (41.0)	1.23 (1.07–1.4)	0.003*
	Yes	95 (46.6)	46 (38.0)	49 (59.0)	–	–
Blood type	Type-O	51 (25.0)	35 (28.9)	16 (19.3)	1.13 (0.97–1.32)	0.119
	Non-O	153 (75.0)	86 (71.1)	67 (80.7)	–	–
CRP (mg/L)	≤ 1.29	45 (22.1)	11 (13.3)	34 (28.1)	1.23 (1.05–1.45)	0.012*
	> 1.29	159 (77.9)	72 (86.7)	87 (71.9)	–	–
RBC (×10^12^/L)	≤ 4.3	71 (34.8)	37 (30.6)	34 (41.0)	0.9 (0.78–1.03)	0.127
	> 4.3	133 (65.2)	84 (69.4)	49 (59.0)	–	–
HGB (g/L)	≤ 137.5	109 (53.4)	69 (57.0)	40 (48.2)	1.09 (0.95–1.25)	0.216
	> 137.5	95 (46.6)	52 (43.0)	43 (51.8)	–	–
HCT (%)	≤ 36.55	32 (15.7)	14 (11.6)	18 (21.7)	0.83 (0.69–1.00)	0.051
	> 36.55	172 (84.3)	107 (88.4)	65 (78.3)	–	–
MCV (fl)	≤ 90.65	93 (45.6)	60 (49.6)	33 (39.8)	1.1 (0.96–1.26)	0.168
	> 90.65	111 (54.4)	61 (50.4)	50 (60.2)	–	–
MCH (pg)	≤ 30.75	124 (60.8)	79 (65.3)	45 (54.2)	1.12 (0.97–1.28)	0.113
	> 30.75	80 (39.2)	42 (34.7)	38 (45.8)	–	–
MCHC (g/L)	≤ 341.5	174 (85.3)	109 (90.1)	65 (78.3)	1.25 (1.04–1.51)	0.020*
	> 341.5	30 (14.7)	12 (9.9)	18 (21.7)	–	–
RDW.CV (%)	≤ 13.4	164 (80.4)	107 (88.4)	57 (68.7)	1.35 (1.15–1.6)	< 0.001*
	> 13.4	40 (19.6)	14 (11.6)	26 (31.3)	–	–
HR	≤ 2.875	43 (21.1)	16 (13.2)	27 (32.5)	0.76 (0.64–0.89)	0.001*
	> 2.875	161 (78.9)	105 (86.8)	56 (67.5)	–	–
RETR (%)	≤ 0.985	24 (11.8)	11 (9.1)	13 (15.7)	0.86 (0.7–1.06)	0.154
	> 0.985	180 (88.2)	110 (90.9)	70 (84.3)	–	–
RET (×10^9^/L)	≤ 82.4	158 (77.5)	90 (74.4)	68 (81.9)	0.90 (0.77–1.06)	0.207
	> 82.4	46 (22.5)	31 (25.6)	15 (18.1)	–	–
WBC (×10^9^/L)	≤ 6.16	124 (60.8)	81 (66.9)	43 (51.8)	1.17 (1.02–1.34)	0.030*
	> 6.16	80 (39.2)	40 (33.1)	40 (48.2)	–	–
NEUT (×10^9^/L)	≤ 2.6	83 (40.7)	58 (47.9)	25 (30.1)	1.19 (1.04–1.37)	0.011*
	> 2.6	121 (59.3)	63 (52.1)	58 (69.9)	–	–
LYMPH (×10^9^/L)	≤ 1.905	132 (64.7)	71 (58.7)	61 (73.5)	0.86 (0.74–0.98)	0.030*
	> 1.905	72 (35.3)	50 (41.3)	22 (26.5)	–	–
MONO (×10^9^/L)	≤ 0.535	119 (58.3)	79 (65.3)	40 (48.2)	1.19 (1.03–1.36)	0.015*
	> 0.535	85 (41.7)	42 (34.7)	43 (51.8)	–	–
PLT (×10^9^/L)	≤ 207.5	68 (33.3)	34 (28.1)	34 (41.0)	0.87 (0.75–1.00)	0.056
	> 207.5	136 (66.7)	87 (71.9)	49 (59.0)	–	–
MPV (fl)	≤ 11.25	150 (73.5)	99 (81.8)	51 (61.4)	1.29 (1.11–1.5)	0.001*
	> 11.25	54 (26.5)	22 (18.2)	32 (38.6)	–	–
P-LCR (%)	≤ 37.85	164 (80.4)	107 (88.4)	57 (68.7)	1.35 (1.15–1.6)	< 0.001*
	> 37.85	40 (19.6)	14 (11.6)	26 (31.3)	–	–
PLR	≤ 196.47	23 (11.3)	10 (8.3)	13 (15.7)	0.84 (0.68–1.03)	0.102
	> 196.47	181 (88.7)	111 (91.7)	70 (84.3)	–	–
NLR	≤ 1.79	92 (45.1)	67 (55.4)	25 (30.1)	1.28 (1.12–1.46)	< 0.001*
	> 1.79	112 (54.9)	54 (44.6)	58 (69.9)	–	–
LMR	≤ 3.64	61 (29.9)	22 (18.2)	39 (47.0)	0.72 (0.62–0.83)	< 0.001*
	> 3.64	143 (70.1)	99 (81.8)	44 (53.0)	–	–
CA19-9 (U/mL)	−	146 (71.6)	109 (90.1)	37 (44.6)	1.72 (1.51–1.95)	< 0.001*
	+	58 (28.4)	12 (9.9)	46 (55.4)	–	–
CA242 (U/mL)	−	170 (83.3)	117 (96.7)	53 (63.9)	1.77 (1.5–2.08)	< 0.001*
	+	34 (16.7)	4 (3.3)	30 (36.1)	–	–
CEA (μg/L)	−	184 (90.2)	120 (99.2)	64 (77.1)	1.83 (1.48–2.26)	< 0.001*
	+	20 (9.8)	1 (0.8)	19 (22.9)	–	–

^*^P < 0.05 was considered statistically significant. CRP, C-reactive protein; RBC, red blood cell; HGB, hemoglobin; HCT, red blood cell specific volume; MCV, mean corpuscular volume; MCH, mean corpuscular hemoglobin; MCHC, mean corpuscular hemoglobin concentration; RDW.CV, red blood cell volume distribution width; HR, hemoglobin-red blood cell distribution width ratio; RETR, reticulocyte ratio; RET, reticulocyte; WBC, peripheral white blood cells; NEUT, neutrophils; LYMPH, lymphocyte; MONO, monocyte; PLT, platelet; MPV, mean platelet volume; P-LCR, platelet-larger cell ratio; PLR, lymphocyte-monocyte ratio; NLR, neutrophil-lymphocyte ratio; LMR, lymphocyte-monocyte ratio; CA19-9, carbohydrate antigen 19-9; CA242, carbohydrate chain antigen 242; CEA, carcinoembryonic antigen; OR, odds ratio; CI, confidence interval; +, CA199 > 37 U/ml, CA242 > 20 U/ml, CEA > 5 μg/L.

The above independent risk factors did not significantly differ between the development cohort and the external validation cohort (**Table 2**).

**Table 2:** Differences between the development cohort and validation cohort

Variable	Level	Overall (%)	Development cohort	Validation cohort	*P*
No. patients		290	204	86	
Age (years)	≤ 55	117 (40.3)	87	30	0.218
	> 55	173 (59.7)	117	56	
Location	Body/tail	164 (56.6)	111	53	0.258
	Head/neck	126 (43.4)	93	33	
Boundary	Clear	144 (49.7)	100	44	0.739
	Blurred	146 (50.3)	104	42	
Blood type	Type-O	79 (27.2)	51	28	0.187
	Non-O	211 (72.8)	153	58	
MCHC (g/L)	≤ 341.5	241 (83.1)	174	67	0.125
	> 341.5	49 (16.9)	30	19	
NLR	≤ 1.79	132 (45.5)	92	40	0.825
	> 1.79	158 (54.5)	112	46	
CA19-9 (U/mL)	≤ 37	209 (72.1)	146	63	0.770
	> 37	81 (27.9)	58	23	
CEA (μg/L)	≤ 5	256 (88.3)	184	72	0.117
	> 5	34 (11.7)	20	14	

### Development of the nomogram

**Figure 3:**
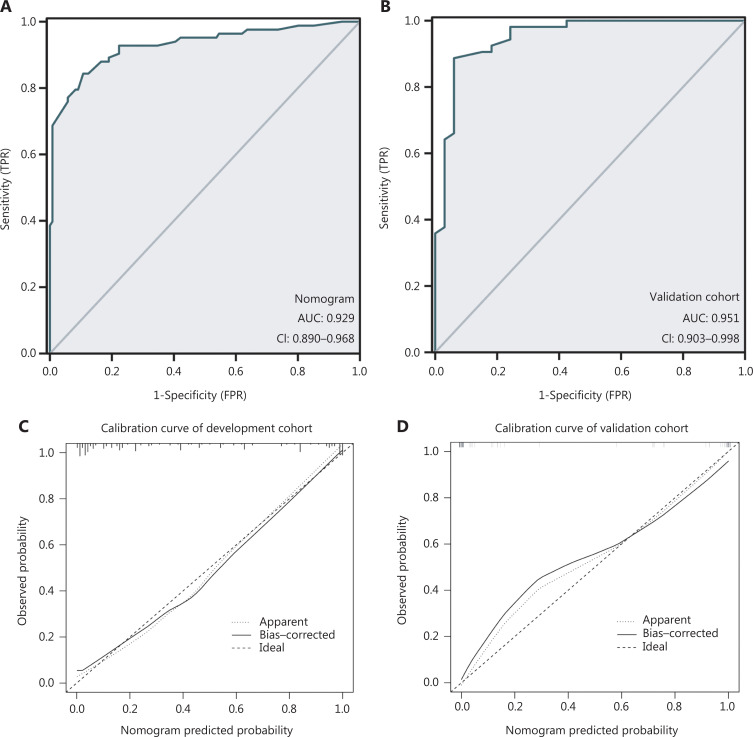
ROC analysis of the malignancy prediction ability of the sum of scores. (A) Internal validation of the nomogram. (B) External validation of the nomogram. (C, D) Nomogram calibration in the model group (C) and the validation group (D). The horizontal axis indicates the risk of malignant PCN predicted by the nomogram, and the vertical axis is the probability of malignant PCN for a random sample of 1000 actual observations. The line from the lower left to the upper right corner of the plot region represents the reference line of the ideal prediction.

The 8 independent predictors described above were integrated into a malignant PCN risk nomogram (**Figure 2B**). To facilitate use, the nomogram is also provided as a calculator online (https://wangjunjinnomogram.shinyapps.io/DynNomapp/). The sensitivity, specificity, and accuracy of prediction of malignant PCN in the development cohort were 84.3%, 89.3%, and 87.3%, respectively. The C-index for the nomogram in the development cohort was 0.929 (95% CI 0.890–0.968), and the Hosmer–Lemeshow goodness of fit test indicated that the model had a good fit (*P* = 0.20). The bootstrap method was used to resample the development cohort 1000 times and plot the calibration curve. The calibration curve indicated excellent agreement between the predicted risk of malignant PCN and the actual observations (**Figure 3A**). The total score for each patient was calculated on the basis of the nomogram, and the optimal cutoff value for the total score in the model cohort, on the basis of pROC, was determined to be 192.5.

In the external validation cohort, the C-index for the nomogram was 0.951 (95% CI 0.903–0.998), and the sensitivity, specificity, and accuracy of predicting malignant PCN were 93.9%, 84.9%, and 88.4%, respectively; the calibration curve also showed good agreement (**Figure 3B**).

### Diagnostic value according to the nomogram cutoff for malignancy

**Figure 4:**
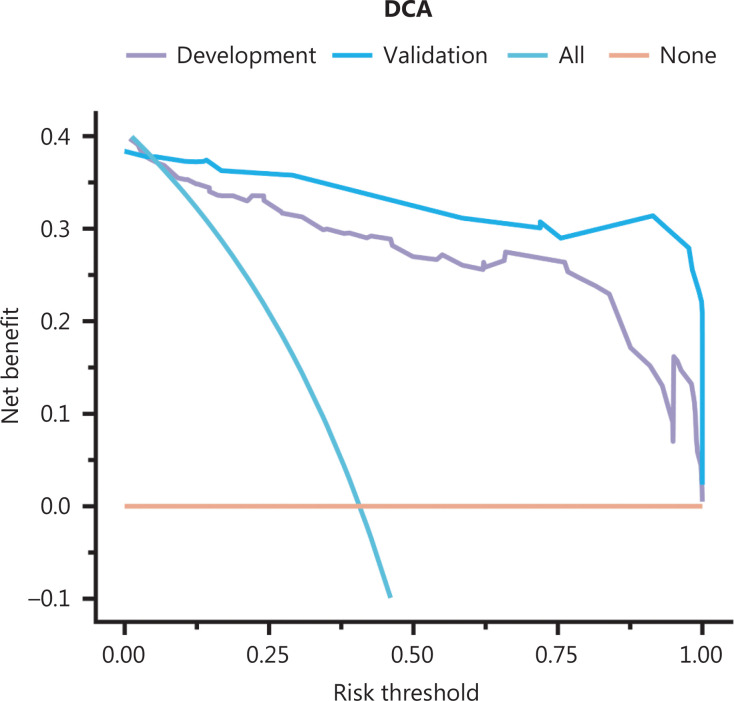
DCA curves for assessing the net benefit of model application in the model development cohort and validation cohort.

After reclassification with the nomogram, the NRI for the model group was 0.268 (*P* < 0.001), and the IDI was 0.271 (*P* for improvement < 0.001) (**Table 3**). To use the nomogram for treatment decision-making, we performed DCA (**Figure 4**). The DCA indicated the net clinical benefit of risk stratification for patients with the model. When the risk threshold probability of the model is > 0.1, the utilization of the model yields more benefits than all-treatment and no-intervention. In our study, treatment refers to other diagnostic examinations and surgery. Surgery is recommended for all patients with substantial symptoms and high suspicion of malignant PCN, whereas patients in whom the nature of PCN cannot be determined and no clinical symptoms are present can be considered for further treatment options after prediction with this model.

**Table 3:** Risk stratification and restratification

Imaging and tumor marker model	Nomogram risk model		Total	Reclassified as higher risk (%)	Reclassified as lower risk (%)
Risk	< 0.5	≥ 0.5			
< 0.5					
No. of patients	117	33	150	33 (22%)	NA
No. of benign	105	7	112	7 (6.25%)	NA
No. of malignant	12	26	38	26 (68.4%)	NA
≥ 0.5					
No. of patients	4	50	54	NA	4 (7.4%)
No. of benign	3	6	9	NA	3 (33.3%)
No. of malignant	1	44	45	NA	1 (2.2%)
Total					
No. of patients	121	83	204	33 (22%)	4 (7.4%)
No. of benign	108	13	121	7 (6.25%)	3 (33.3%)
No. of malignant	13	70	83	26 (68.4%)	1 (2.2%)

NRI = 26.8%; *P* < 0.001.

IDI = 0.2708; *P* < 0.001.

## Discussion

In this study, the preoperative testing results and demographic characteristics of the model cohort were analyzed in conjunction with postoperative pathological data, and 8 factors—increased CA19-9, increased CEA, increased NLR, increased MCHC, non-O blood group, age > 55 years, cyst located in the head or neck of the pancreas, and unclear cyst imaging border—were finally identified as independent predictors of malignant PCN. A predictive model was developed accordingly, and the area under the ROC curve was calculated to be 0.929 (95% CI 0.890–0.968); the calibration curve showed approximate agreement with the ideal diagnostic curve. The risk prediction model based on the above independent risk factors performed well in the validation group, with a diagnostic accuracy of 88.4%. We also applied the model to 4 newly admitted patients who were not included in the developmental cohort for preoperative scoring to predict their malignant risk and validated the results with their postoperative pathological results. The information on the patients and the predicted results are shown in **Supplementary Figures S1–S4**. In addition, this model included basic clinical data, imaging information, and laboratory examinations of patients, thus enabling better understanding and providing greater reliability than the current guidelines and recommendations, as well as better discriminatory power. When the above risk factors are present simultaneously, the model indicates a higher risk of malignancy of this tumor, and surgery can be considered. However, the number of samples included in this study was small, and the possibility that the accuracy was elevated because of selection bias cannot be excluded. In future studies, we plan to subsequently include more specimens for model development and prospective validation against this model.

Currently, 3 guidelines provide recommendations on PCN surveillance and surgical resection based on symptoms and known risk of malignancy: the 2015 American Gastroenterological Association (AGA)^18,19^, the International Association of Pancreatology (IAP)^20^, and the European Study Group on Cystic Tumours of the Pancreas (European)^21^. The IAP^20^ and the European^21^ guidelines were revised in 2017 and 2018, respectively. The decision to surgically remove PCN depends on an accurate diagnosis. Current guidelines for predicting the risk of malignancy in pancreatic cysts require expensive imaging and invasive examinations. Cystic fluid analysis is used to supplement the information obtained from imaging. Although cyst classification based on tissue biopsy and cystic fluid analysis is more accurate, it may carry some risks and complications, owing to the invasive character of the procedure. In addition, single-site biopsies cannot interpret the entire lesion, thus increasing the probability of missed diagnosis. Using imaging for classification is noninvasive. CT and MRI have equivalent accuracy, but CT is associated with a risk of repeated exposure to radiation, whereas MRI is the recommended modality for diagnosis and surveillance of PCN, owing to its high resolution and ability to discriminate the main pancreatic duct. Unfortunately, these imaging techniques are expensive and have limited accuracy in distinguishing malignant from benign cysts.

In a study with a 5-year follow-up, the baseline weighted prevalence of pancreatic cysts was 49.1% among 1,077 observed participants. In the 367 participants who received 5-year follow-up without cysts at baseline, the incidence of pancreatic cysts was 12.9% (2.6% per year)^1^, and a significant association was observed between prevalence and age. Older people had a higher prevalence of PCN. Although screening and intervention for patients with PCN, a group with high pancreatic cancer risk, is necessary, the prevalence of PCN leads to thousands of unnecessary medical and surgical interventions every year. These procedures are associated with substantial risks and comorbidities, particularly for older people. Even in patients who do not require surgery, substantial time and resources are needed to monitor patients for several years after initial detection. This surveillance includes expensive imaging techniques and invasive examinations, such as repeat endoscopic ultrasound-guided fine-needle aspiration biopsy, and eventual resection, thus increasing the financial burden on the health care system. In recent years, with the development of imaging, the accurate localization of abdominal ultrasound has become important for the diagnosis of pancreatic cancer, because it has the advantages of easy operation, higher accuracy, low cost, non-invasiveness, and wide applicability to the population. Simultaneously, abdominal ultrasound is the preferred imaging modality for almost all patients during outpatient visits or physical examinations. In addition, choosing ultrasound does not require abandoning other examinations. Ultrasound was used only as a screening index in our model; if the ultrasound findings were contrary to the predicted results of the model, we recommended that patients undergo further examinations^22^. In addition, although cyst size and solid components of the cyst were not included in the model in the results of this study, we believe that these factors remain important in the preoperative assessment of benignity and malignancy, because SCN and MCN are rich in cystic fluid and appear as large cystic foci, but have a low malignant tendency; therefore, tumor diameter is often not an accurate predictor of PCN benignity or malignancy. In a meta-analysis including 1058 patients, the ratio (OR) of IPMN with a diameter > 3 cm to potential malignancy was 62.4 (30.8–126.3) and was found to be the strongest predictor.

In this study, we combined abdominal ultrasound, routine blood tests, serum tumor markers, and demographic characteristics of patients to develop a new preoperative stratified prediction model for PCN, which can be used for the first consultation and long-term monitoring of patients with high accuracy and safety, thus providing economic benefits.

A meta-analysis investigating the ability of serum CA19-9 and CEA to identify invasive and malignant IPMN has found that CA19-9 has a sensitivity of 52% and 40% and a specificity of 88% and 89%, whereas CEA has a sensitivity of 18% and a specificity of 95% and 93% for invasive and malignant IPMN^23^. CA19-9 is commonly used for clinical monitoring of PDAC progression and assessment after surgical resection, and CEA has been used as a biomarker for various gastrointestinal malignancies^24^.

SCN often appears in the pancreatic body and tail, mostly in middle-aged women. In contrast, IPMN occurs mostly in the pancreatic head of older male patients, and MCN occurs mostly in older women. IPMN has been found to have a relatively higher malignant potential^25,26^. In our results, sex could not be considered a predictive factor for malignant PCN, but the location of the cyst in the pancreatic head and neck was an independent risk factor, in agreement with the cystic characteristics described above. One study has reported that patients with malignant cysts are significantly older than those with benign cysts^27^. This result is also consistent with our finding that age is associated with malignant PCN.

Inflammation plays a key role in cancer occurrence and progression^28^. Recently, extensive evidence has indicated that inflammatory indicators also play an important role in predicting benign and malignant PCN^29^. Our study provided another demonstration that NLR is an independent factor influencing malignant PCN; the threshold value selected for NLR was 1.79, in general agreement with the previous literature^29^. The significant correlation between increased NLR and malignant PCN may be based on the ability of neutrophils to recruit and activate inflammatory cells through the production of cytokines and chemokines, which in turn act on the tumor microenvironment.

One study^30^ has shown that non-O blood types have a higher risk of pancreatic cancer than O blood types (OR = 1.44, 95% CI 1.144-1.82), and individuals with A and B blood types have a significantly higher frequency of PDAC than those with O blood types. Patients with O blood types more often have well-differentiated PDAC than do those with non-O blood types, whereas patients with AB blood types more often exhibit poorly differentiated tumors. These findings are consistent with the results of the present study. The relationship between blood group and malignancy has been partially explored. The ABO gene is located on chromosome 9, and a genomic study of pancreatic cancer and normal populations has found a significant association between ABO genetic locus variants on chromosome 9q34 and pancreatic cancer (*P* < 0.001)^31^. The ABO gene does not directly encode ABO antigen but encodes a glycosyltransferase that mediates mucin-type glycosylation. Aberrant mucin-type glycosylation (hereafter O-glycosylation) is a typical feature of the malignant transformation of epithelial cells. Tumor-associated aberrant O-glycans (Tn and T antigens) are detected in most PDACs and are structurally associated with blood group A and B glycans^32^. The presence of base deletions in the O allele results in a loss of glycosyltransferase activity in the end product^33^. ABO blood group IgM lectins are associated with PDACs, and O-GalNAc modifications in PDACs are associated with the presence of O-glycans. O-GalNAc-modified glycoproteins in PDAC may influence cancer pathogenesis. ABO blood group antigens can alter the host inflammatory response, thereby leading to malignant tumor progression and spread^34^; they can also alter intercellular and cell-extracellular matrix interactions, thereby promoting tumor development^35^. MCH, MCV, MCHC, Hb, and HCT are the most widely and commonly used markers reflecting nutritional and anemia conditions; among these, MCH, MCV, and MCHC are commonly used to rapidly evaluate anemia type. Mean corpuscular hemoglobin concentration (MCHC), an indicator of the average hemoglobin concentration per red blood cell, is sensitive to the assessment of tumor progression, angiogenesis, and metastasis of neoplastic cells. Studies^36,37^ have shown that in patients with hepatorenal syndrome in decompensated cirrhosis, increased MCHC indicates poor prognosis. MCHC is closely associated with tumor size and prognosis, and in oral tumors, MCHC increases significantly with tumor enlargement. MCH and MCHC reflect rapid compensatory regeneration of the bone marrow caused by hemolysis and anemia. Male patients with high MCHC have a significantly higher risk of developing prostate cancerr^38^, in agreement with our findings that high MCHC is an independent risk factor for malignant PCN. The mechanisms involved in the pathological elevation of MCHC and tumorigenesis are not well understood, but may possibly be a consequence of the long-term effects of iron-dependent oxidative stress on erythrocyte structure and cancer pathogenesis^39^. In addition, in some types of cancer, such as renal cancer, hepatocellular carcinoma, or ovarian cancer, a non-compensatory increase in erythropoietin due to tumors can also lead to increased MCHC.

This research has several limitations. First, it was a retrospective analysis including only cases with postoperative pathology suggestive of PCN, thus potentially leading to selection bias. In the future, we plan to use the model in prospective clinical studies to further validate its efficacy. In addition, the sample was limited in this study, and only one center was selected for external validation; consequently, the sample representativeness is relatively low, and more samples must be included for model validation.

## Conclusions

In conclusion, this study provides clinicians with a simple, effective, and non-invasive predictive model for benign and malignant PCN, which facilitates the consultation, management, and treatment of patients with PCN. Studies on PCN are increasing, and preoperative risk factors for predicting malignant PCN are a major area of research. For clinicians, a comprehensive preoperative analysis of various risk factors, accurate weighing of surgical risks and malignancy risks, precise determination of surgical indications, and the development of intervention plans that minimize invasion and maximize efficacy according to the specific conditions of different patients will bring the greatest benefit to patients with PCN.
