# Supplementary material for: Preoperative ultrasound combined with routine blood tests in predicting the malignant risk of pancreatic cystic neoplasms
Source: Cancer Biol Med. 2022 Nov 1;19(10):1503–16. doi: 10.20892/j.issn.2095-3941.2022.0258 (PMC9630525; doi:10.20892/j.issn.2095-3941.2022.0258)
Supplement: Supplementary file 1 [file cbm-19-1503-s001.pdf]

Supplementary materials

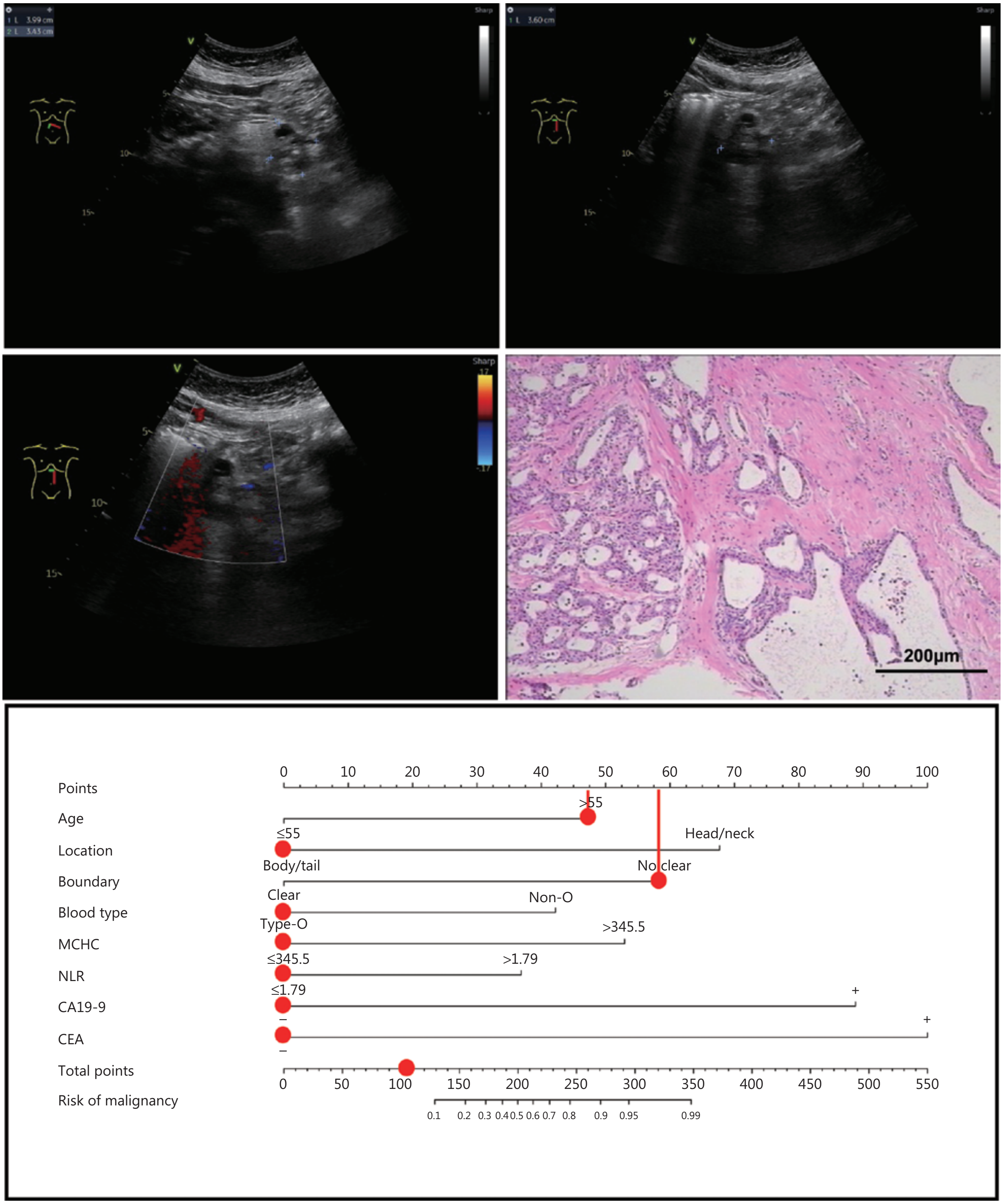

Figure S1 Female, 59 years old; ultrasound shows a cystic mass in the tail of the pancreas with poorly defined, irregular borders, and strong central echogenic calcification. O type blood, CA19-9: 11.53 U/ml, CEA: 1.47 μg/L, MCHC: 334 g/L, NLR: 1.57. The model suggested benignity, and the postoperative pathology indicated SCN.

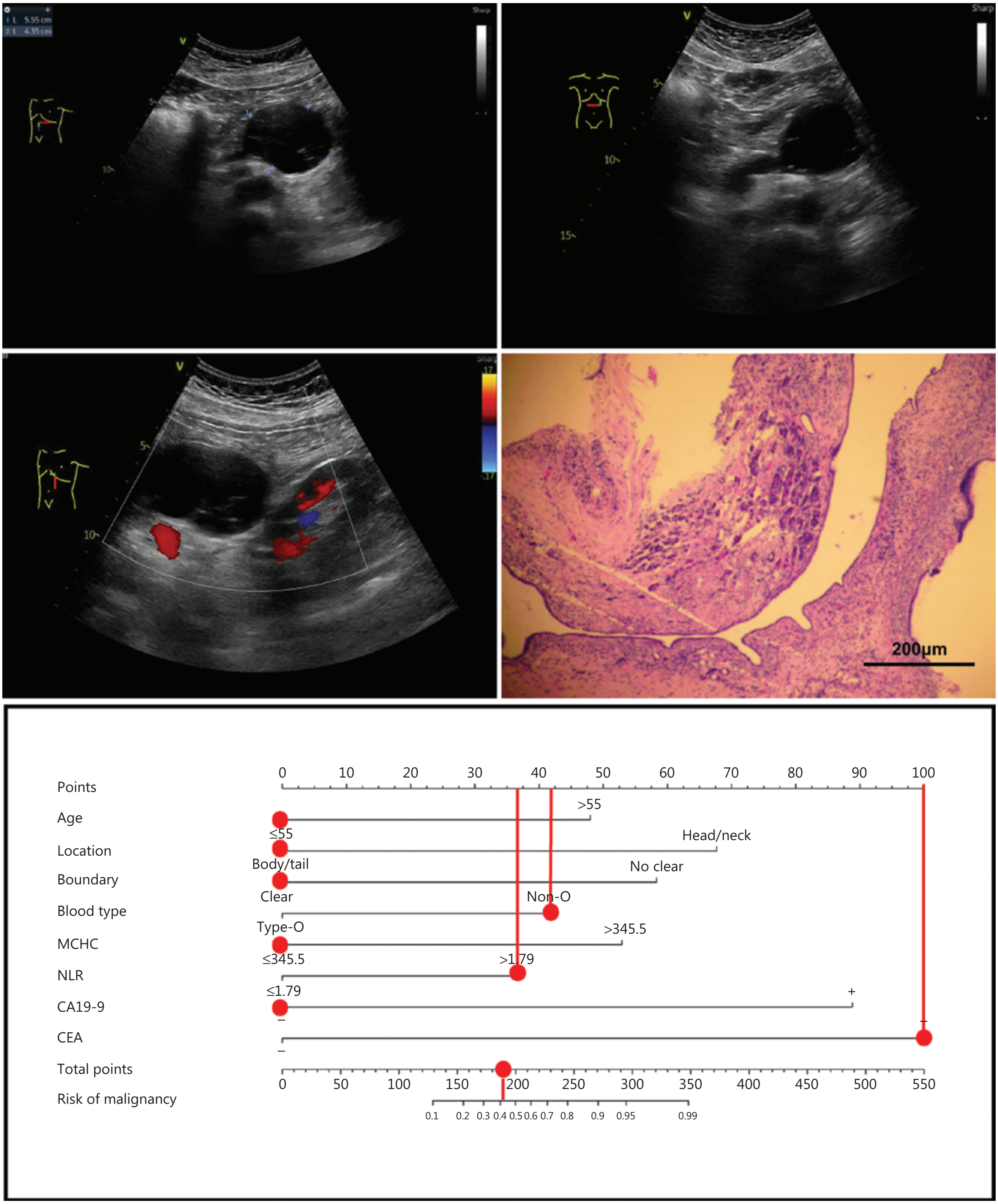

Figure S2 Female, 40 years old; ultrasound shows a cystic mass in the body and tail of the pancreas with clear borders, regular morphology, visible multiple fibrous separations, and no significant dilatation of the pancreatic duct. A type blood, MCHC: 341 g/L, NLR: 3.05, CA19-9: 24.19 U/ml, CEA: 10.53 μg/L. The model suggested benignity, and the postoperative pathology indicated MCN.

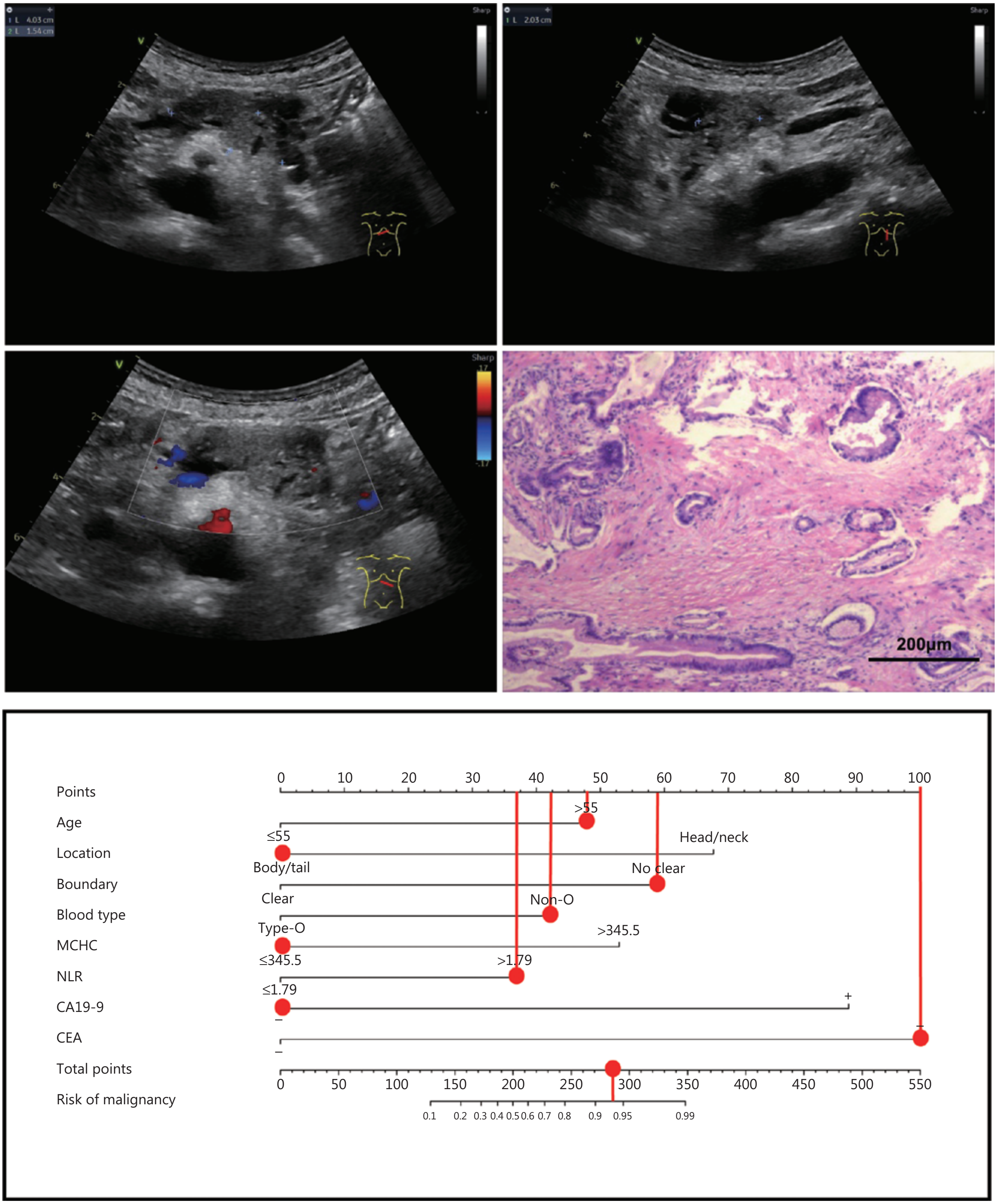

Figure S3 Female, 63 years old; ultrasound shows a middle-low echo reflection area with poorly defined, irregular, and heterogeneous echogenicity in the pancreatic duct located in the tail and body of the pancreas. A type blood, CA19-9: 7.13 U/ml, CEA: 9.50 μg/L, MCHC: 331 g/L, NLR: 2.17. The model suggested malignancy, and the postoperative pathology indicated IPMN with associated invasive carcinoma.

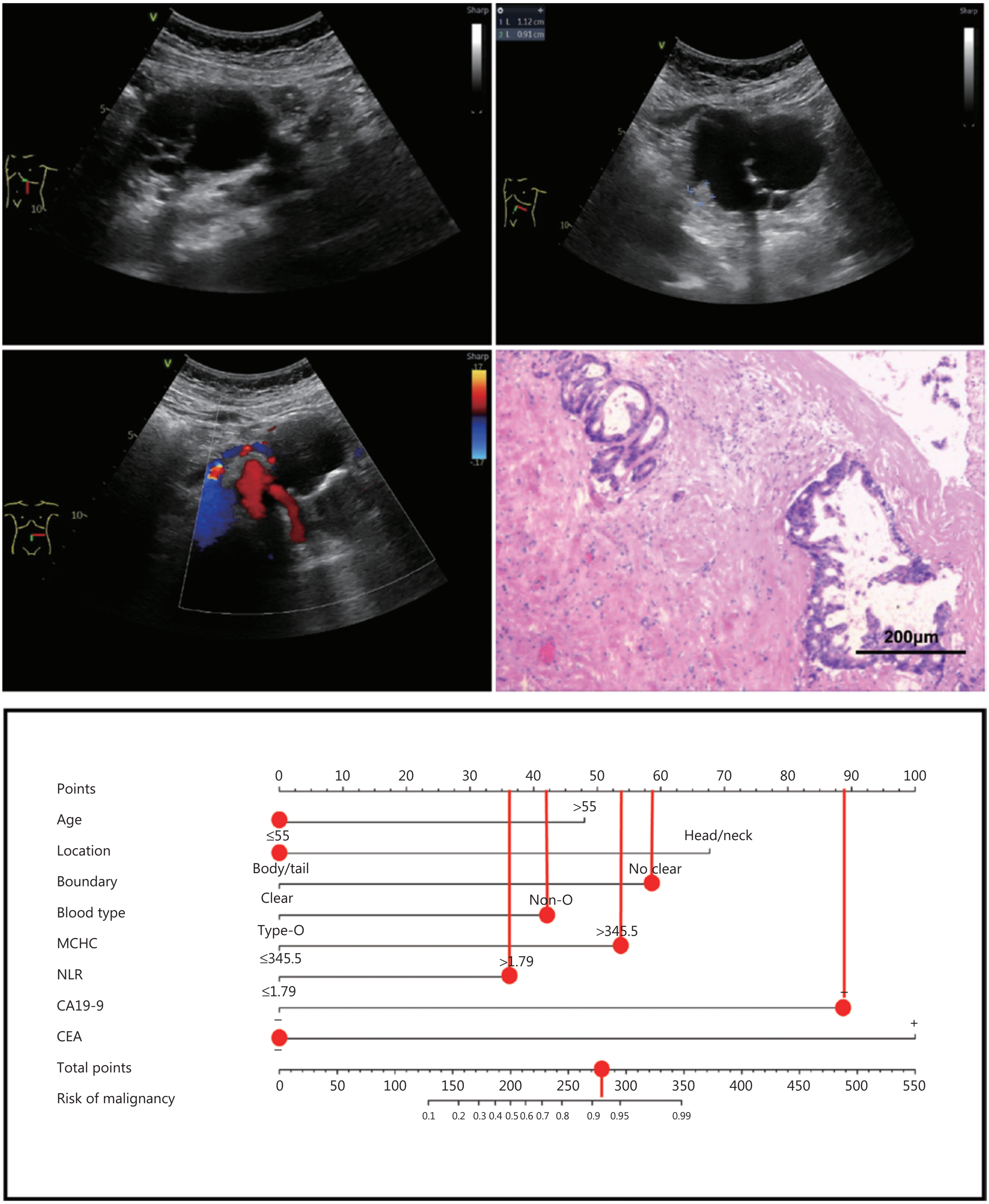

Figure S4 Female, 50 years old; ultrasound showed a cystic mass in the body and tail of the pancreas with an unclear border, irregular morphology, thick local cystic wall, and mural nodule with multiple strong echogenic separations and calcifications. B type blood, MCHC: 353 g/L, NLR: 2.98, CA19-9: 313.9 U/ml, CEA: 2.44 μg/L. The model suggested malignancy, and the postoperative pathology indicated moderately differentiated ductal adenocarcinoma with mucinous adenocarcinoma.
